# Cytotoxicity of Newly Synthesized Quinazoline–Sulfonamide Derivatives in Human Leukemia Cell Lines and Their Effect on Hematopoiesis in Zebrafish Embryos

**DOI:** 10.3390/ijms23094720

**Published:** 2022-04-25

**Authors:** Ali S. Alqahtani, Mostafa M. Ghorab, Fahd A. Nasr, Mohammad Z. Ahmed, Abdullah A. Al Mishari, Sabry M. Attia, Muhammad Farooq Khan

**Affiliations:** 1 Department of Pharmacognosy, College of Pharmacy, King Saud University, P.O. Box 2457, Riyadh 11451, Saudi Arabia; mahmed4@ksu.edu.sa; 2 Medicinal, Aromatic and Poisonous Plants Research Center, College of Pharmacy, King Saud University, P.O. Box 2457, Riyadh 11451, Saudi Arabia; aalmshari@ksu.edu.sa; 3 Department of Drug Radiation Research, National Center for Radiation Research and Technology (NCRRT), Egyptian Atomic Energy Authority (EAEA), Nasr City, Cairo 11371, Egypt; mmsghorab@yahoo.com; 4 Department of Pharmacology and Toxicology, College of Pharmacy, King Saud University, P.O. Box 2457, Riyadh 11451, Saudi Arabia; attiasm@ksu.edu.sa; 5 Bioproducts Research Chair, Department of Zoology, College of Science, King Saud University, P.O. Box 2455, Riyadh 11451, Saudi Arabia; fmuhammad@ksu.edu.sa

**Keywords:** apoptosis, quinazoline–sulfonamide, leukemia cells, zebrafish

## Abstract

Many quinazoline derivatives with pharmacological properties, such as anticancer activity, have been synthesized. Fourteen quinazoline derivatives bearing a substituted sulfonamide moiety (**4a–n**) were previously synthesized and fully characterized. These compounds exerted antiproliferative activity against cell lines derived from solid tumors. Herein, the antileukemic activities of these compounds (**4a–n**) against two different leukemia cell lines (Jurkat acute T cell and THP-1 acute monocytic) were investigated. Our investigation included examining their activity in vivo in a zebrafish embryo model. Remarkably, compounds **4a** and **4d** were the most potent in suppressing cell proliferation, with an IC_50_ value range of 4–6.5 µM. Flow cytometry analysis indicated that both compounds halted cell progression at the G2/M phase and induced apoptosis in a dose-dependent manner. RT-PCR and Western blot analyses also showed that both compounds effectively induced apoptosis by upregulating the expression of proapoptotic factors while downregulating that of antiapoptotic factors. In vivo animal toxicity assays performed in zebrafish embryos indicated that compound **4d** was more toxic than compound **4a**, with compound **4d** inducing multiple levels of teratogenic phenotypes in zebrafish embryos at a sublethal concentration. Moreover, both compounds perturbed the hematopoiesis process in developing zebrafish embryos. Collectively, our data suggest that compounds **4a** and **4d** have the potential to be used as antileukemic agents.

## 1. Introduction

According to the GLOBOCAN estimate of 2020 global cancer statistics, leukemia caused around 311,594 deaths and is expected to cause approximately 474,519 new cases [1]. Leukemia is a group of hematological malignancies that influence white blood cells and can be acute or chronic according to the basis of cell maturity [2]. Leukemia can be treated using a variety of medications; however, the search for safe and more effective drugs to reduce the negative consequences of such therapies remains a considerable challenge [3,4]. Thus, looking for novel drugs to treat various kinds of leukemia still represents an interesting field in cancer therapy.

**Figure 1:**
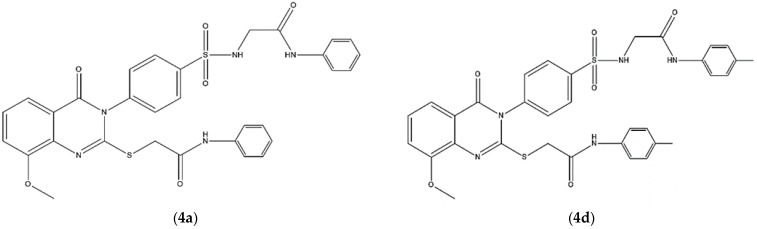
Chemical structure of 2-(4-(8-Methoxy-4-oxo-2-((2-oxo-2-(phenylamino) ethylthio) quinazolin-3(4*H*)-yl) phenylsul-fonamido)-*N*-phenylacetamide (**4a**) and 2-(4-(8-Methoxy-4-oxo-2-((2-oxo-2-(*p*-tolylamino) ethylthio) quinazolin-3(4*H*)-yl) phenylsul-fonamido)-*N*-(*p*-tolyl) acetamide (**4d**).

Quinazoline is a biologically important scaffold with various pharmacological effects, including antiviral, anti-inflammatory and anticancer activities [5]. The field of quinazoline sulfonamide synthesis is continuously being researched, and only a few research groups have revealed their tentative successes in synthesizing quinazolines with sulfonamide functionality in recent decades [6]. In the field of chemotherapeutic agents, quinazoline and sulfonamide moieties have been recognized as compounds with a remarkable therapeutic effect against various tumors [6,7]. Further research on this class has also led to the discovery of numerous compounds, such as erlotinib and lapatinib, that have effective anticancer activity via cell cycle arrest and apoptosis induction [8,9]. In addition, quinazoline scaffolds have also served to develop both apoptosis inducers and inhibitors [10]. As a continuation of our earlier study [11], this work aimed to explore the antileukemic activity of two previously reported compounds—**4a** (2-(4-(8-Methoxy-4-oxo-2-((2-oxo-2-(phenylamino) ethylthio) quinazolin-3(4*H*)-yl) phenylsul-fonamido)-*N*-phenylacetamide) and **4d** (2-(4-(8-Methoxy-4-oxo-2-((2-oxo-2-(*p*-tolylamino) ethylthio) quinazolin-3(4*H*)-yl) phenylsul-fonamido)-*N*-(*p*-tolyl) acetamide) (Figure 1)—against two different leukemia cell lines (Jurkat and THP-1 cells). We also further examined the effects of compounds **4a** and **4d** in a zebrafish model. Common problems that can arise during the late stages of drug development in cell-based drug screening include drug toxicity and side effects resulting from the off-target binding of the drug; such obstacles can be easily overcome by using a whole animal model chemical screening approach. The zebrafish embryo is an excellent whole animal screening model, which allows for a preliminary drug screening to be carried out without the need for extensive labor and time. Zebrafish embryos have been successfully used as a very powerful hematopoiesis animal model. Hematopoietic cells can be directly observed in zebrafish embryos because they are transparent, models of human blood diseases can be modeled in zebrafish embryos and small-molecule screens can reveal new drugs to treat human disease [12,13,14]. Therefore, to verify the in vitro biological activity of the active compounds (**4a** and **4d**) in this study, their activity against malignant hematopoiesis in zebrafish embryos was used as an in vivo drug screening model.

## 2. Results and Discussion

### 2.1. Effects of Synthesized Compounds on Leukemia Cell Viability

The synthesis of quinazoline carrying biologically active substituted benzene sulfonamide moieties **4a**–**n** was described in our previous work [11]. In our previous study, these compounds were examined on various cell lines derived from solid tumors and promising findings were reported [11]. As a result, we assumed that they would have similar potent activity against leukemia cells. Indeed, promising results against leukemic cells were observed in this study. Jurkat and THP-1 cell lines were treated with increasing doses of the synthesized compounds for 24 h and cell viability was determined using an MTT assay. The IC_50_ values for the effective compounds were calculated from the dose–response curve. The cytotoxicity assay results (Table 1) showed that only six (**4a**, **4b**, **4d**, **4f**, **4h** and **4j**) out of fourteen compounds exerted antiproliferative activity against the tested leukemia cells. Based on the obtained IC_50_ values, it was found that compounds **4a** and **4d** exhibited the uppermost activity. The Jurkat and THP-1 cell lines were equally sensitive to compounds **4a** and **4d**, with superior activity being observed for compound **4d**. In general, compound **4d** was more active than **4a** against both cell lines. These results align with our previous report, in which compound **4d** displayed the most potent activity [11].

Compared to the cytotoxicity results from the previously tested solid tumor cell lines [11], we found that compound **4a** showed a more potent cytotoxic activity against leukemia cells in terms of IC_50_ values. Additionally, compound **4a** exhibited less cytotoxicity in normal HUVEC cells [11], suggesting a selective cytotoxic activity towards leukemia cell lines. On the other hand, compound **4d** exerted a relatively close cytotoxic activity against solid and leukemic cells. Moreover, the IC_50_ values of compound **4d** reported in this study (4.4 and 4.2 µM) were comparable to those of doxorubicin (positive control), as summarized in Table 1.

A preliminary structure activity relationship (SAR) analysis focused on the impact of the hydrophobic and electronic nature of the substituent used in this study. In addition, it was focused on the impact of the type and length of the linkers used and the distal moieties. From the obtained results, it was found that compound **4a** (IC_50_ = 6.1 and 6.5 µM) and compound **4d** (IC_50_ = 4.2 and 4.6 µM) showed a promising cytotoxic activity against the tested leukemia cancer cell line. In general, quinazoline derivative **4d**, with a hydrophobic electron-donating (+inductive [+]) 8-methoxy and hydrophobic electron-donating (+I) 4-methyl group at the 2- and 3-position, respectively, showed higher activity than the unsubstituted phenyl distal moiety **4a**.

### 2.2. Compounds ***4a*** and ***4d*** Caused G_2_/M Cell Cycle Arrest

**Figure 2:**
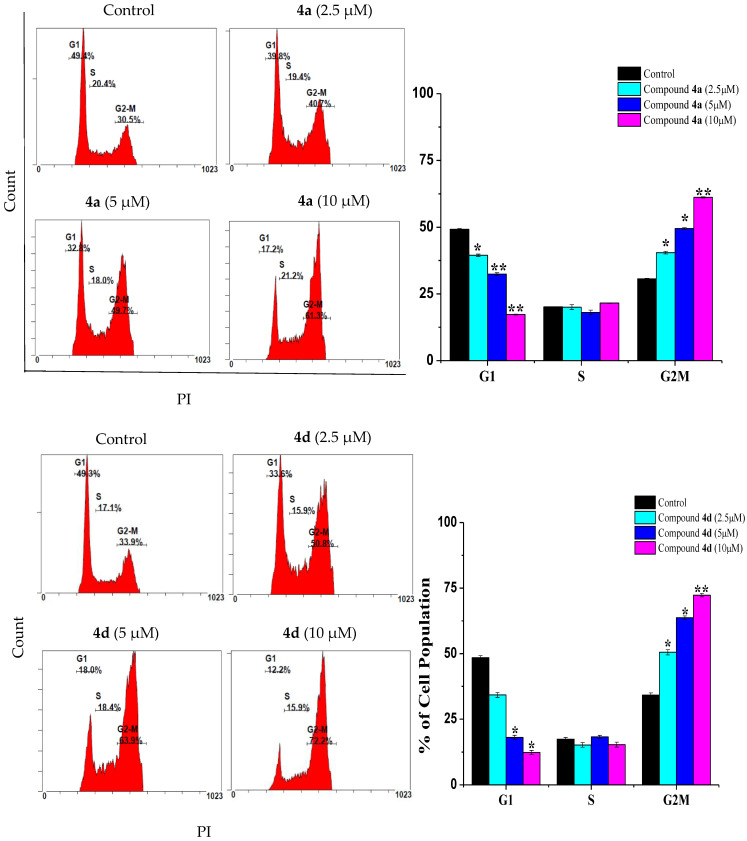
Effect of compounds **4a** and **4d** on the cell cycle progression of Jurkat cells. Jurkat cells were cultivated in the presence of 2.5, 5 and 10 µM of compounds **4a** and **4d**, while untreated cells served as a control. Data are presented as the means ± SD of three experiments. * *p* < 0.05 and ** *p* < 0.01, compared with the control group.

**Figure 3:**
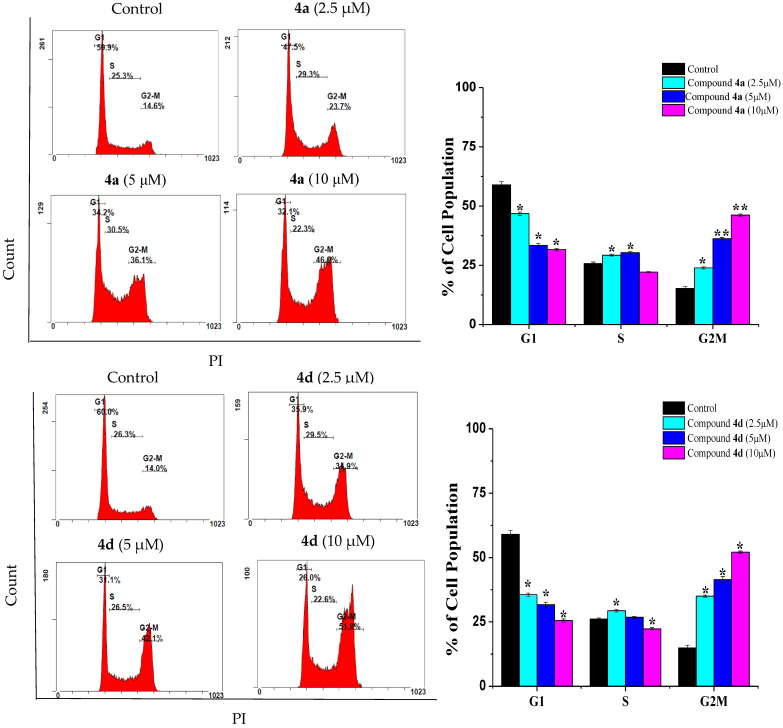
Effect of compounds **4a** and **4d** on cell cycle distribution of THP-1 cancer cells. Cells were treated with 2.5, 5 and 10 μM of compounds **4a** and **4d** for 24 h, while untreated cells served as a control. Data are presented as the means ± SD of three experiments. * *p* < 0.05 and ** *p* < 0.01, compared with the control group.

Cell cycle arrest is a crucial mechanism by which anticancer drugs suppress cancer cell proliferation [15]. Therefore, the prevention of cell cycle progression is considered as an important goal in cancer treatment. As shown in Figure 2, the incubation of Jurkat cells for 24 h with various doses of **4a** (2.5, 5 and 10 µM) led to a significant increase in the population of cells at G_2_/M (40.45 ± 0.49%, 49.50 ± 0.28% and 61.15 ± 0.21%, respectively). As with **4a**, the incubation of Jurkat cells with compound **4d** resulted in a significant increment in the G_2_/M cell population (50.5 ± 1%, 63.7 ± 0.68% and 72.3 ± 0.64%) compared to the control cells (34.2 ± 0.82%). Similarly, the exposure of THP-1 cells to compound **4a** (Figure 3) resulted in a significant accumulation of cells at the G_2_/M phase (23.9 ± 0.48%, 36.25 ± 0.55% and 46.2 ± 0.48% compared to 15.25 ± 0.77% in control cells). In the same manner, THP-1 cells treated with 2.5, 5 and 10 µM of compound **4d** increased the cell population at the G_2_/M phase to 35 ± 0.44%, 41.45% ± 1.14 and 52.1 ± 0.42% of the control cell value. Our data is in line with Zhang et al. (2021), who found that quinazoline derivatives caused a G2/M cell cycle arrest in H1975 lung cancer cells [7]. In the same study, a similar effect was also reported for the drug gefitinib. Moreover, the scenario described here for G2/M cell cycle arrest in both Jurkat and THP-1 cells was also previously reported with doxorubicin for the same cells, where the cells responded mainly by G2/M arrest [16,17,18].

### 2.3. Compounds ***4a*** and ***4d*** Inhibited Leukemic Cancer Cell Proliferation through Apoptosis Induction

Flow cytometry was also employed to assess the impact of compounds **4a** and **4d** on cell death by detecting phosphatidylserine exposure on the outer membrane of apoptotic cells. Apoptosis stimulation is a major objective in the development of antineoplastic drugs. In fact, most of the currently utilized anticancer drugs in clinical oncology trigger cancer cell death through apoptotic signaling pathways [19,20].

**Figure 4:**
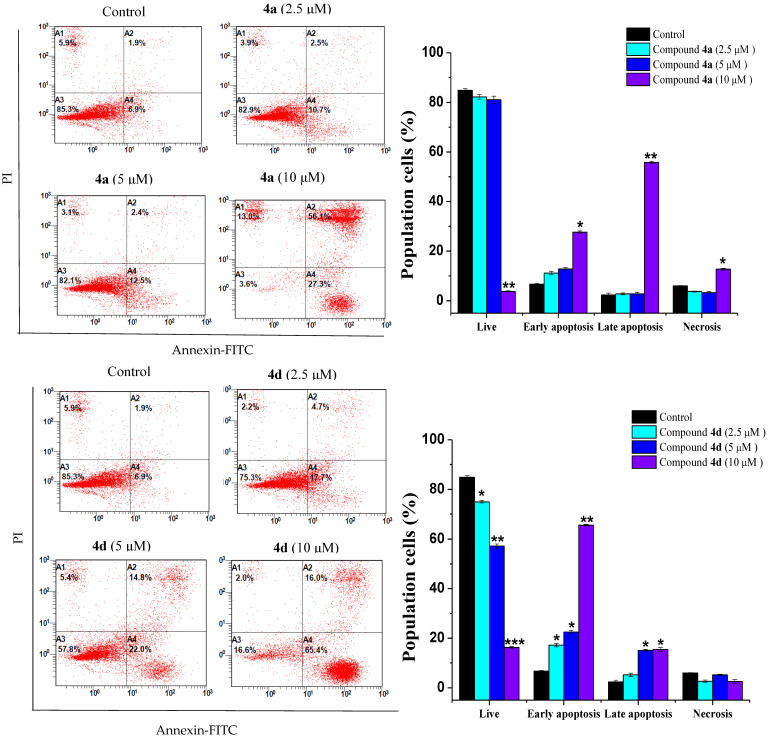
Jurkat leukemic cancer cell apoptosis induced by compounds **4a** and **4d**. Jurkat cells were treated with each compound (2.5, 5 and 10 μM) and stained with Annexin-FITC-PI, followed by FACS analysis. * *p* < 0.05, ** *p* < 0.01 and *** *p* < 0.001, compared with the control group.

**Figure 5:**
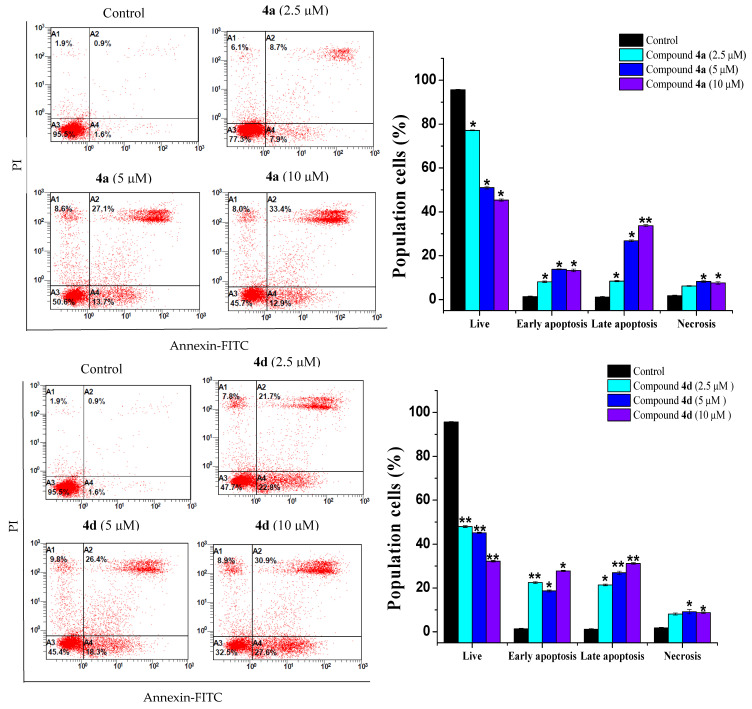
Effect of compounds **4a** and **4d** on Jurkat and THP-1 cell apoptosis. Cells were incubated with each compound at 2.5, 5 and 10 µM for 24 h. The level of apoptosis was assessed by flow cytometry using the Annexin V-FITC-PI staining. Data are representative of three experiments. * *p* < 0.05 and ** *p* < 0.01, compared with the control group.

The Jurkat and THP-1 cell lines were treated with different concentrations of both compounds. In both cell lines, the treatments resulted in apoptotic cell death (Figure 4 and Figure 5). The Jurkat cell line showed the best apoptotic response to both compounds **4a** and **4d**. The percentage of early apoptotic cells in 4a-treated Jurkat cells increased from 6.7 ± 0.28% in the control cells to 11.2 ± 0.71%, 12.85 ± 0.49% and 27.6 ± 0.49% at 2.5, 5 and 10 µM, respectively (Figure 4). A remarkable increase in late apoptotic cells was also observed with 10 µM of compound **4a**. Similarly, the percentage of early apoptotic cells in Jurkat cells treated with compound **4d** increased progressively from 6.7 ± 0.28% to 17.2 ± 0.71%, 22.5 ± 0.71% and 65.65 ± 0.35 after treatment with the indicated concentrations (Figure 4). In addition, the THP-1 cells exhibited a dose-dependent increase in the number of early and late apoptotic cells after treatment with both compounds **4a** and **4d** at the indicated concentrations (Figure 5). The scenario described here is in line with several previous studies that have documented the inhibition of cancer cell proliferation via apoptosis promotion by quinazoline derivatives [10,21].

**Figure 6:**
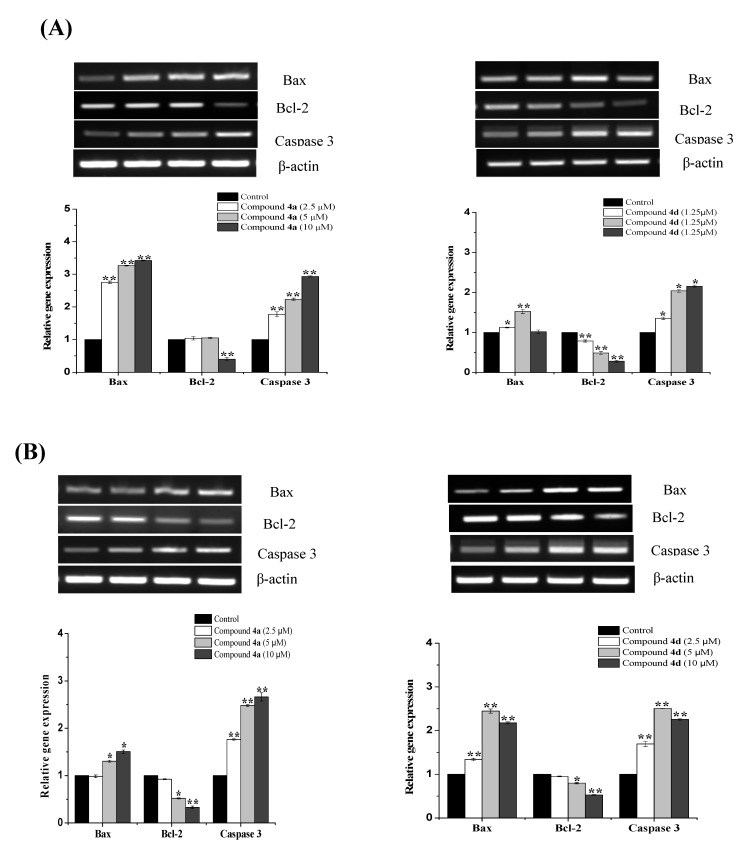
RT-PCR analysis of the apoptosis-related genes Bax, Bcl-2 and caspase-3 in (**A**) Jurkat and (**B**) THP-1 cell lines after 24 h of treatment with compounds **4a** and **4d**. The expression levels of the target genes were normalized against β-actin expression and expressed as a fold change relative to the control samples. * *p* < 0.05 and ** *p* < 0.01 indicate a significant difference in comparison to the untreated control.

Next, the expression levels of apoptotic markers were examined using RT-PCR and Western blot analysis to investigate the mechanism of apoptosis generated by target compounds. Deregulation of the BCL-2 family genes is now recognized as a common event in many types of cancer, and it is likely that targeting this family will be a useful addition to current cancer therapies [22]. Thus, the changes in the mRNA levels of Bax and Bcl-2, as proapoptotic and antiapoptotic members, respectively, were simultaneously analyzed. As expected, the treatment of the cells with both compounds resulted in an increase in the expression of Bax and a decrease in the expression of Bcl-2. As shown in Figure 6A, the treatment of Jurkat cells with compound **4a** for 24 h significantly increased the expression levels of Bax (2.75-, 3.27- and 3.42-fold) and caspase-3 (1.77-, 2.23- and 2.93-fold) in comparison to the untreated cells, while a significant decrease in the expression levels of Bcl-2 was only detected with the 10 µM treatment (0.4-fold; *p* < 0.01). On the other hand, treatment with compound **4d** slightly enhanced the levels of Bax by 1.12-, 1.52- and 10.2-fold and markedly increased the expression of caspase-3 by 1.35-, 2.04- and 2.15-fold, respectively. Similarly, the exposure of THP-1 cells to compound **4a** resulted in a significant increase in the expression of Bax with the 5 and 10 µM treatments (1.31- and 1.51-fold, respectively) and a significant downregulation of Bcl-2 at the same concentration (0.52- and 0.33-fold, respectively). The expression levels of caspase-3 were also noticeable after the exposure of THP-1 cells to various concentrations of each compound (Figure 6B).

**Figure 7:**
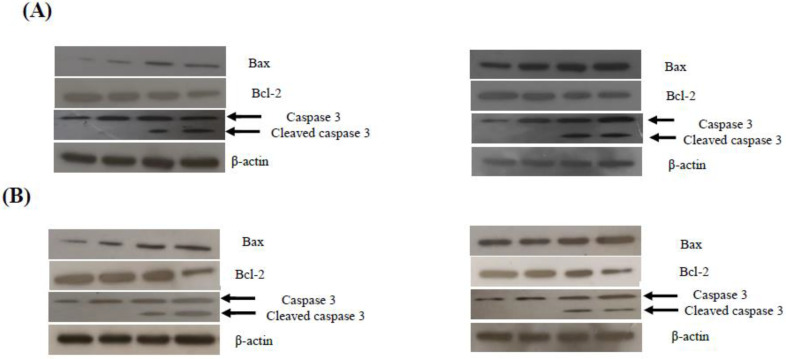
Effects of compounds **4a** and **4d** on the expression of apoptotic-related proteins in (**A**) Jurkat cells and (**B**) THP-1 cells. Both cells were treated with each compound for 24 h and a Western blot analysis was performed. Protein from each sample was resolved and β-actin was used as an internal control.

In regard to the protein levels, we also found that both compounds altered the expression of apoptotic-related proteins—Bax expression was increased, while Bcl-2 expression was decreased (Figure 7). The involvement of executioner caspase-3, which is cleaved during apoptosis initiation, was also evaluated. Indeed, the detection of cleaved caspase-3 is considered as a credible marker for cells undergoing apoptosis [23]. As demonstrated in Figure 7A,B, both compounds caused an increase in the cleaved form of caspase-3 in treated Jurkat and THP-1 cells, which further indicates that **4a** and **4d** mediate apoptosis in leukemia cells.

### 2.4. Determination of the In Vivo Toxicity of Compounds ***4a*** and ***4d*** in Zebrafish Embryos

Zebrafish embryos were used in this study to determine the in vivo toxicity of quinazoline–sulfonamide derivatives. Zebrafish embryos are an ideal in vivo drug screening system that share physiological characteristics with mammals; the embryos and juveniles are extremely small in size, allowing for their use in high-throughput screens. Moreover, their rapid embryonic development and sufficiently developed organs can enable the early detection of toxicities [24]. Most newly synthesized anti-cancer drugs are abandoned during the development process because these molecules possess toxicities on normal cells as well. Embryonic development results from normal (non-cancer) cell proliferation and division; hence, another purpose of conducting the zebrafish drug screening assays was to assess the toxicity of quinazoline–sulfonamide derivatives toward normal cell proliferation.

**Figure 8:**
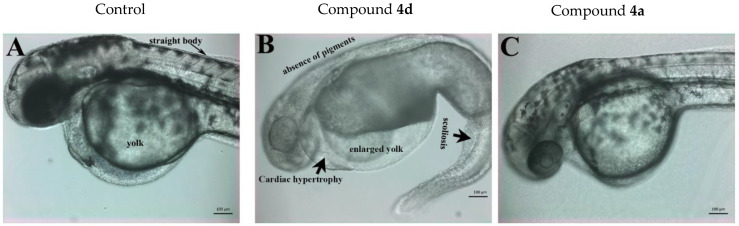
Representative live images of zebrafish embryos at 48 hpf. (**A**) Mock (0.5% *V*/*V*)-treated zebrafish embryos developed normally. The embryos had normal circulation (shown separately in videos) and the body and eyes were fully pigmented. They had a normal yolk size and their bodies were also straight. (**B**) Zebrafish embryos treated with compound **4d** (sublethal dose of 0.76 µM shown here). The embryos showed multiple teratogenic defects in response to exposure to compound **4d**, including scoliosis (bent spine and body), no pigmentation, an enlarged yolk and cardiac hypertrophy. (**C**) The zf embryos treated with compound **4a** (15.88 µM) showed the same phenotype as the mock-treated embryos, with full pigmentation, no sign of cardiac hypertrophy and straight bodies. However, the treated embryos did not have active circulation (shown in supplementary videos). The scale bar represents 100 µm and the images were taken under the same magnification.

The in vivo animal toxicity assay performed in zebrafish embryos indicated that most of the compounds, except compounds **4a** and **4d**, were not active (not toxic) even at the highest concentration used (50 µM). These compounds neither killed the embryos nor induced any teratogenic effect (data not shown). Compounds **4a** and **4d** affected the development of zebrafish embryos. Compound **4d** turned out to be more toxic than compound **4a**; **4d** induced toxicities and its LD50 (the concentration at which 50% of the embryos died) value was just 1.67 µM. Moreover, compound **4d** also induced multiple levels of teratogenic phenotypes in zebrafish embryos at a sublethal concentration (0.3–0.76 µM). The treated embryos showed severe scoliosis, enlarged yolk, cardiac hypertrophy and an absence of pigmentation (Figure 8B).

On the other hand, compound **4a** was less toxic and its LD_50_ value was more than 27 µM. The development, growth and phenotype of zebrafish embryos treated with compound **4d** (7 µM) looked very similar to the control embryos, and no obvious teratogenesis was detected (Figure 8A,C).

### 2.5. Compound ***4a*** Affected the Haemopoiesis Process in Developing Zebrafish Embryos without Inducing Developmental Toxicity

Hematopoiesis is a process occurring during embryonic development that gives rise to blood cells of different lineages. Various blood cell diseases, including leukemia, are the result of abnormalities in this developmental program. In zebrafish, hematopoiesis is very similar to any other vertebrates, and the developmental and genetic programs of hematopoiesis are highly conserved [25]. Zebrafish have been used as a prominent model for human leukemia and related disorders [26,27]. Chemical library screening for leukemic research has been successfully carried out in zebrafish and various hit compounds have been identified through these in vivo screening assays [28,29,30].

The wild type zebrafish embryos were treated with a serial dilution (0.3 to 76 µM) of compound **4a** and **4d** in order to assess the effect on blood cell formation and development. Both compounds affected the hematopoiesis process in developing zebrafish embryos; however, compound **4d** also induced multiple embryonic abnormalities. As shown in the time lapse microscopy results (supplementary videos), the zebrafish embryos that were treated with **4a** at a concentration more than 1.58 µM did not have circulation at 48 hpf, yet these embryos did not show any other embryonic abnormalities. Compound **4d** induced severe toxicity in zebrafish embryos and its activity against blood formation could be due to a secondary effect; hence, compound **4d** was not used in further experiments.

**Figure 9:**
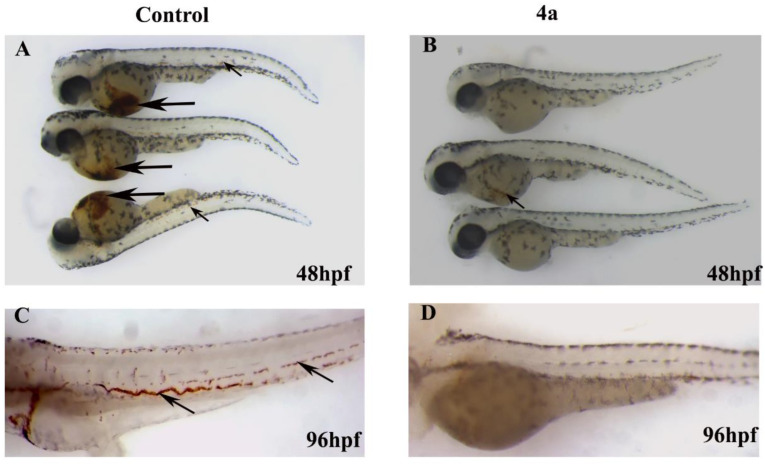
Representative images of zebrafish embryos showing the O-dianisidien staining of erythrocytes. (**A**) Group of three control embryos at 48 hpf showing strong dianisidien-positive cells in the duct of Cuvier, indicated by black arrows, and also in the dorsal aorta and cardinal vein (trunk region by arrowhead). (**B**) Embryos treated with compound **4a** (15.88 µM) did not stain positive for O-dianisidien (top and bottom embryos) or stained very weakly (middle embryo). (**C**) Mock-treated zebrafish embryos stained with O-dianisidien at 96 hpf marked all the erythrocytes (black arrows). (**D**) No positive O-dianisidien staining was detected in zebrafish embryos treated with compound **4a** at 96 hpf.

Erythrocyte development is also another indication of hematopoiesis; thus, O-dianisidien staining was used to stain the erythrocytes in the control and **4a**-treated embryos. The embryos were exposed to **4a** at the shield stage (6 hpf) and fixed at 48 and 96 hpf with O-dianisidien. As shown by the black arrows in Figure 9, the control embryos stained positive for O-dianisidien, especially in the yolk area known as the duct of Cuvier where the anterior cardinal and posterior cardinal veins return blood from the head and trunk, respectively; the two vessels come together side by side in the common cardinal vein that crosses both sides of the body [31]. The treated embryos showed a significant reduction in stained cells (Figure 9B). The effect of **4a** on zebrafish hematopoiesis was also time dependent. Prolonged exposure until 96 h completely abolished hematopoiesis and no hemoglobin staining was observed in 15.88 µM **4a**-treated embryos (Figure 9D).

**Figure 10:**
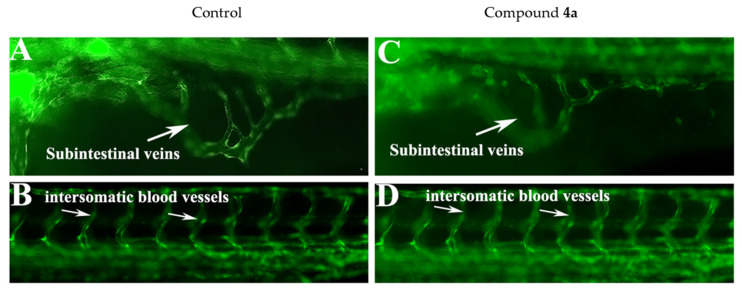
Representative live micrograph of transgenic zebrafish embryos Tg (fli1:EGFP)y1 at 72 hpf showing the effect of compound 4a on blood vessel formation. (**A**–**D**) Control embryos showing the number and structure of sub-intestinal angiogenic blood vessels in the yolk area as well as the intersegmental or intersomatic angiogenic blood vessels. (**C**,**D**) The structure and number of sub-intestinal angiogenic blood vessels in the yolk area and the intersegmental or intersomatic angiogenic blood vessels, which were not affected by exposure to compound **4a**.

During embryonic development, the hematopoietic (which make various blood cells) and endothelial cells (which make blood vessels) differentiate from common hemangioblasts [32]. In order to verify whether compound **4a** had any effect on blood vessel formation, the development of blood vessels was monitored in the zebrafish transgenic line TG (Fli1: EGFP), which expresses green fluorescent protein in the blood. The transgenic embryos were treated with **4a** (1.58 µM) at the shield stage (6 hpf) and the effect on blood vessels was evaluated at 72 hpf. The formation of intersomatic blood vessels (isv) and sub-intestinal veins (sib) were investigated in the treated embryos. The results are shown in Figure 10. It can be seen that **4a** did not affect the number or structures of blood vessels in the treated embryos.

## 3. Conclusions

Among all the newly synthesized compounds, only **4a** and **4d** induced a significant level of cytotoxicity in leukemia cell lines, and **4d** was the most toxic compound; however, the in vivo study suggested that compound **4d** induced toxicity in normal cell proliferation during zebrafish embryonic development. For this reason, compound **4a** was concluded to be a favorable anti-leukemia target molecule, as it was the least toxic toward normal cells while still being toxic toward leukemia cell lines and hematopoiesis in zebrafish embryos. Flow cytometry analysis demonstrated that both compounds caused G_2_/M cell cycle arrest and induced apoptotic cell death. In vivo data revealed that **4a** disrupted hematopoiesis in less than the micro molar range without inducing gross teratogenicity or toxicity. Hematopoietic defects were observed in embryos treated with **4a** at a concentration between 0.2 and 1.00 μM, whereas more than 17 μM was required to induce lethality or toxicity in zebrafish embryos. In general, the presented data offer new insights into the development of quinazoline–sulfonamide derivatives as potential antileukemic agents.

## 4. Materials and Methods

### 4.1. Compounds

A series of quinazoline compounds containing a biologically active substituted sulfonamide at the 3-position and substituted aromatic moieties at the 2-position, including phenyl **4a**, 2-methyl phenyl **4b**, 3-methyl phenyl **4c**, 4-methyl phenyl **4d**, 2-ethyl phenyl **4e**, 3-ethyl phenyl **4f**, 4-ethyl phenyl **4g**, 4-methoxy phenyl **4h**, 4-ethoxy phenyl **4i**, 3,5-dimethoxy phenyl **4j**, 3,4,5-trimethoxy phenyl **4K**, 2-methyl-4-nitro phenyl **4l**, 2-methyl-6-nitro phenyl **4m** and 2,4-dinitro phenyl **4n**, were detailed and characterized as the compounds **4a**-**n** in our previous work [11]. The structure of the synthesized compounds was characterized by microanalysis, FT-IR, ^1^H-NMM, ^13^C-NMR and mass spectral data.

### 4.2. Cell Lines and Cell Viability Assay

Human acute T-cell leukemia (Jurkat, Clone E6-1) and THP-1 human monocytic (ATCC-TIB202) cells were grown at 37 °C in a humidified incubator (Panasonic, Moriguchi, Osaka, Japan) with 5% CO_2_ and maintained in RPMI 1640 media (Gibco, Rockville, MD, USA) supplemented with 10% heat-inactivated fetal bovine serum and 1% penicillin/streptomycin (Gibco, Rockville, MD, USA).

For the detection of the viability of Jurkat and THP-1 cells, an MTT assay was employed as previously described [33]. Briefly, both Jurkat and THP-1 cells in the logarithmic growth phase were seeded at a density of 1 × 10^5^ cells/well on a 24-well plate and treated with various concentrations (40, 20, 10, 5 and 2.5 µM) of each compound. After the incubation period (24 h), the MTT reagent, (Invitrogen, Thermo Fisher Scientific, Waltham, MA, USA) (5 mg/mL) was added to each well. Then, the plates were incubated for 4 h at 37 °C and the formazan was dissolved in DMSO (Sigma-Aldrich, St. Louis, MO, USA). Thereafter, the optical density was measured at 570 nm using an ELISA reader (Bio-Tek, Elx-800, Winooski, VT, USA) and cell viability was calculated according to the equation % V = A − Ao/Ac − Ao × 100. The IC_50_ (compound concentration required for the inhibition of 50% of cells) values were calculated from the dose–response curves using the OriginPro 8.5 (OriginLab Corporation, Northampton, MA, USA).

### 4.3. Cell Cycle Analysis

Both Jurkat and THP-1 cells were cultured in a 12-well plate and were treated with different concentrations of compounds **4a** and **4d**. After a 24 h incubation time, the cells were washed with 1× phosphate-buffered saline (PBS), fixed with 70% ice-cold ethanol and kept for 4 h at 4 °C. Thereafter, the cells were precipitated, washed with 1x PBS and resuspended in 500 μL of PBS containing propidium iodide (BioLegend, San Diego, CA, USA; PI; 50 μg/mL) with 0.1 mg/mL of Ribonuclease A and further incubated for 30 min in the dark. The analysis of the cell cycle was performed using flow cytometry (Cytomics FC 500; Beckman Coulter, Brea, CA, USA) and CXP software V. 3.0.

### 4.4. Detection of Apoptosis by Flow Cytometry

After treatment with compounds **4a** and **4d** (2.5, 5 and 10 µM) for 24 h, apoptotic cells were detected using an apoptosis detection kit according to the supplier’s instructions (BioLegend, San Diego, CA, USA). In brief, both untreated and treated cells were collected, washed with PBS and then resuspended in 0.1 mL Annexin binding buffer(1x). Thereafter, the cells were stained with Annexin V-FITC and PI (5 µL each) for 15 min in the dark. The samples were then quantified by a FACS Scan Flow Cytometer (Cytomics FC 500; Beckman Coulter, CA, USA).

### 4.5. Extraction of RNA and Reverse Transcription Polymerase Chain Reaction (RT-PCR) Analysis

RT-PCR was used to assess the level of mRNA in proportion to β-actin, the housekeeping gene. An RNA stabilization reagent (Qiagen, Germantown, MD, USA), was used to extract total RNA from treated and untreated THP-1 and Jurkat cells, and the RNA was quantified by measuring the absorbance at 260 nm (Thermo Fisher Scientific, Waltham, MA, USA). Using the Superscript IV Vilo Master mix (Invitrogen; Thermo Fisher Scientific, Waltham, MA, USA), a total of 1 µg of RNA from each group was utilized to synthesize complementary DNA (cDNA). The cDNA samples were kept at a temperature of −20 degrees Celsius. Bax, Bcl2, caspase-3 and β-actin mRNA assays were performed with RT-PCR using the newly generated cDNA. An aliquot of 1 µL of cDNA from each group was amplified in a 25 µL reaction mixture with 5X FIREpol master mix (Solis BioDyne, Tartu, Estonia). The oligonucleotide sequences were designed using Integrated DNA Technologies (IDT). The forward and reverse primers for Bax were 5′-TTTGCTTCAGGGTTTCATC-3′ and 5′-ATCCTCTGCAGCTCCATGTT-3′, respectively. The primers for Bcl2 were 5′-TGATGCCTTCTGTGAAGCAC-3′ and 5′-ACAGGCGGAGCTTCTTGTAA-3′; 5′-TGGAATTGATGCGTGATGTT-3′ and 5′-GGCAGGCCTGAATAATGAAA-3 for caspase-3; and 5′-CATCGTGATGGACTCTGGTG-3′ and 5′-TTTGATGTCACGCACGATTT-3′ for β-actin. The sample was denatured for 5 min at 95 °C, then amplified using 32 cycles of denaturation at 95 °C for 30 s, annealing at 55 °C for 60 s and elongation at 72 °C for 60 s, followed by final elongation at 72 °C for 5 min using a SimpliAmp Thermal Cycler (Applied Biosystems, Foster City, CA, USA). After that, a 1.2 percent agarose gel containing ethidium bromide was used to electrophorese 20 µL of the PCR-amplified material, and the bands on the gel were photographed.

### 4.6. Western Blotting

The THP-1 and Jurkat cells were treated with different concentration of each compound for 24 h, and untreated cells were taken as a control. After 24 h of treatment, cells were harvested and washed twice with 1× PBS, and the harvested cells were lysed by incubation in a lysis buffer (20 mM Tris-HCl (PH 7.5), 0.5 M NaCl, 1 mM EDTA, 1 mM EGTA, 0.25% Triton X-100, protease inhibitor mixture, 2 mM PMSF and 1 mM DTT) for 2 h on ice. The obtained lysate was clarified by centrifuging at 1300 rpm at 4 °C for 15 min. The protein content of the supernatant was measured using a Bradford protein assay (Bio-Rad, Hercules, CA, USA). The expression levels of Bax, Bcl2, caspase-3 and β-actin were detected with a Western blot by resolving 25 µg of protein on 12% SDS-PAGE. After electrophoresis, the proteins on the gel were transferred to a PVDF membrane (Bio-Rad, CA, USA) and the membrane was blocked with 5% BSA for 1h at room temperature, followed by washing with Tris-buffered saline, 0.1% Tween-20 (TBST) and incubation with primary antibodies (Santa Cruz Biotechnology, Inc., Santa Cruz, CA, USA; 1:2000) against Bax, Bcl2, caspase-3 and β-actin overnight at 4 °C. β-actin was used as an internal control. Following this, an incubation with horseradish peroxidase-conjugated secondary antibodies diluted to 1:4000 in PBS containing 5% BSA was completed at room temperature for 30 min. The binding of immunoproteins was detected using an enhanced chemiluminescent assay (ECL; Amersham, UK) and X-ray films (Amersham, UK).

### 4.7. In Vivo Zebrafish Study

#### 4.7.1. Animal Treatment

Wild type (AB Tubingen) and transgenic “Tg (Fli-1: EGFP)” [34] zebrafish were raised and maintained in the animal facility of the Bioproducts Research Chair, College of Science, Department of Zoology, King Saud University, Riyadh, Saudi Arabia following the guidelines described in [35].Embryos were obtained via natural pairwise mating by setting the pair of adult fish a day before the spawning in one litter breeding tanks in which male and female were separated by divider and kept. The fertilized embryos were syphoned using sterile plastic pasture pipets and cultured in an air incubator at 28.5 °C.

#### 4.7.2. Preparation of Compounds and Embryo Treatments

The compounds were dissolved in molecular biology grade DMSO (Sigma Aldrich) to prepare a stock solution of 25 mM. DMSO (0.5% (*V*/*V*)) was used as a mock control. Embryos at identical developmental stages were sorted at the shield stage, transferred to 35 mm sterile glass dishes and exposed to a serial dilution (0.1–10 µM) of each compound in a total volume of 5 mL of embryo medium. The embryos remained exposed to the compound until the end of the experiment (3 days), where the embryo medium, including the compound, was changed daily. The embryonic mortality and teratogenic effects were monitored every 24 h until the end of the experiment. The treatment was repeated at least three times (biological repeats). The LC_50_ values for zebrafish embryonic toxicity were calculated using an updated Probit analysis [36]. The embryos used in this study were less than 5 days post-fertilization; thus, we were not required to obtain approval for the procedure from the institutional animal care and use committee as described in [37].

#### 4.7.3. Hemoglobin Staining of Live Zebrafish Embryos

To determine whether the quinazoline–sulfonamide derivatives affected blood formation in zebrafish embryos, O-dianisidien dye was used to stain red blood cells. The control and treated embryos were grown to the 20-somite stage; then, phenylthiourea (catalog # P7629, Sigma Aldrich) was added to the embryo medium. The embryos were cultured and fixed at 48 and 72 h post-fertilization (hpf) for O-dianisidien staining. The staining was performed in the dark for 30 min at room temperature within a solution containing O-dianisidine (0.6 mg/mL), 0.01 M sodium acetate (PH 4.5), 0.65% hydrogen peroxide and 40% ethanol. Once stained, the embryos were washed with distilled water and fixed in 4% paraformaldehyde for 1 h at room temperature. The embryos were washed with phosphate-buffered saline (PBS) containing 0.1% Tween-20.

### 4.8. Statistical Analysis

All data are represented as the mean ± standard deviation (SD). Statistical significance was determined by a paired t-test using the OriginPro software. Statistical significance was defined as a *p* value of less than 0.05.

## Figures and Tables

**Table 1:** IC_50_ values of compounds **4a–n** against Jurkat and THP-1 leukemia cells.

Compound No.	Cell Lines and IC_50_ (µM)	
	Jurkat	THP-1
**4a**	6.1 ± 0.19	6.5 ± 0.18
**4b**	17.4 ± 0.28	16.7 ± 0.61
**4c**	-	-
**4d**	4.4 ± 0.2	4.2 ± 0.2
**4e**	-	-
**4f**	12.55 ± 0.63	11.9 ± 0.3
**4g**	-	-
**4h**	13.4 ± 0.15	9.75 ± 0.35
**4i**	33.8 ± 1.5	8.37 ± 0.1
**4j**	-	-
**4k**	-	-
**4l**	-	-
**4m**	-	-
**4n**	-	-
Doxorubicin	1.3 ± 0.01	1.1 ± 0.01

(-) Inactive. Values are represented as the average ± SD (*n* = 3).

## Data Availability

The data presented in this study are available on request from the corresponding author.

## Supplementary Materials

The following supporting videos can be downloaded at: https://zenodo.org/record/6425147 (accessed on 8 April 2022), time lapse microscopy of zebrafish embryos at 48 hpf; control (mock 0.5% *V*/*V*) showing active circulation and compound **4a** (10 µg/mL)-treated embryos showing absence of hematopoiesis.
